# Analysis of Poyang Lake water balance and its indication of river–lake interaction

**DOI:** 10.1186/s40064-016-3239-5

**Published:** 2016-09-13

**Authors:** Zengxin Zhang, Yuhan Huang, Chong-Yu Xu, Xi Chen, Elica M. Moss, Qiu Jin, Alisha M. Bailey

**Affiliations:** 1 Joint Innovation Center for Modern Forestry Studies, College of Biology and the Environment, Nanjing Forestry University, Nanjing, 210037 China; 2 State Key Laboratory of Water Resources and Hydropower Engineering Science, Wuhan University, Wuhan, 430072 China; 3 Department of Geosciences, University of Oslo, 0316 Oslo, Norway; 4 State Key Laboratory of Hydrology-Water Resources and Hydraulics Engineering, Hohai University, Nanjing, 210098 China; 5 Department of Biological and Environmental Sciences, Alabama A&M University, Normal, AL 35762 USA

**Keywords:** Water balance, Poyang Lake, The Three Gorges Dam, River–lake interaction

## Abstract

In recent years, water shortage is becoming one of the most serious problems in the Poyang Lake. In this paper, the long-term water balance items of the Poyang Lake have been analyzed to reveal the coupling effects of Three Gorges Dam (TGD) and droughts on the water balance of Poyang Lake. The results indicate that: (1) the water balance items of Poyang Lake vary greatly, e.g. lake precipitation and inflow decrease during the past several decades while evaporation and water consumption increase significantly; (2) the water balance of Poyang Lake has been affected by the operation of TGD. Negative lake water balance in recent years leads to a serious water shortage problem in the Poyang Lake. Moreover, the operation of TGD also changed the river–lake relationship in the lower Yangtze River basin; (3) the coupling effects of drought and TGD on the lake water balance has been analyzed by using composite analysis method and it can be found that the operation of TGD has significantly altered the lake water balance. But it is not the only factor that affects the lake water balance, and the droughts might cause their relations to be much more complicated.

## Background

Lakes are important components of the earth’s hydrological cycle providing a variety of services for humans and ecosystem functioning (Kummu et al. 2013; Cao et al. 2016, Yao et al. 2016). Evaluation of water balance and hydrological characteristics in a lake region is important in helping manage water supply and predicting flooding and water shortages (Dessie et al. 2015; Xu et al. 2014; Li et al. 2016a, b; Ye et al. 2016). Lake water balance analysis is one of the key research focuses in the hydrological study (Zhang et al. 2014a; Sene et al. 2016; Li et al. 2016c). The significant potential consequences of climate change and human activities might alter regional hydrological cycles and subsequently change lake water quantity and quality (Kummu et al. 2013; Piao et al. 2010; Prasad et al. 2015). Generally, most of these studies were focused on the impacts of climate change on the lake water balance and they ignored the impacts of human activities on the lake water balance (Dessie et al. 2015). As the change of lake water is caused by climate change and human activities, it is necessary to study the coupling effects of natural and human activities on the lake water balance (Cai et al. 2009). Peters and Buttle (2010) investigated the natural and human induced changes in the Lake Athabasca–Peace–Athabasca Delta and found that the regulated hydrology could produce large stormflow and high lake levels, but only under extreme climatic events in areas below the dam and/or human-induced alterations to normal reservoir operation.

In recent years, abilities of the dams to change natural hydrologic processes have increased in many river basins (Yan et al. 2010; Li et al. 2016b). Particularly, large dams could profoundly alter river flow regime and result in a series of consequences (Gao et al. 2013; Yan et al. 2016; Mei et al. 2016b). For example, the closure of the Aswan Dam completely modified the flow regime in the Nile River, leading to a marked decline in agricultural productivity, accelerated coastal erosion, and increased salt water intrusion (Stanley and Warne 1993). Yan et al. (2010) assessed the effects of dam operation on flow regimes in the lower Yellow River and found that the flow magnitude of Yellow River was much smaller and the high flows were cut as well as postponed temporarily.

As the world’s largest dam, the Three Gorges Dam (TGD), worldwide attention has been focused on how the dam impacts the environment in its downstream (Lian et al. 2013; Stone 2008; Li et al. 2016d). The operation of TGD has caused endless debate in China on its potential impacts on the environment and humans (Lian et al. 2013). The increase in the river–lake water level gradient induced by the TGD altered the lake balance by inducing greater discharge into the Yangtze River, which is probably responsible for the current lake shrinkage (Mei et al. 2015). It has been found change in the timing of wetland emergence in the Poyang Lake during the dry season since the establishment of TGD (Mei et al. 2016a). The TGD may also lead to the Yangtze geomorphological change and induces variations of water discharge in the Poyang Lake (Dai et al. 2014; Mei et al. 2015). As the suspended sediment content and fluxes in the middle and lower reaches of the river decreased noticeably in the early stages after the operation of TGD, the riverbed has turned from depositional before the dam construction to erosional afterwards (Dai and Liu 2013).

The recent droughts in the Yangtze River basin coinciding with the operation of the TGD have also drawn people’s attention to the water shortage problem. It aroused a debate over whether the TGD contributed to the decrease in water level of the Poyang Lake (Lai et al. 2014). These problems are believed to be induced by climate anomalies and dam regulation. The water shortage in the Poyang Lake basin could be explained by changes of inputs and outputs of water balance in the Poyang Lake basin. In the lake basin, the changing trends of water balance are basically consistent with the effect of temperature and precipitation, lake outflow during July to September (Xu et al. 2014). In addition to the basin effect (basin discharge generated by rainfall), the TGD operation has affected the Yangtze River discharge and water level (Guo et al. 2012), which further influences water exchange between the Poyang Lake and the Yangtze River. Jiang and Huang (1996) pointed out that the TGD has changed the characteristics of streamflow in the middle and lower Yangtze River. Hu et al. (2007) inferred that the Yangtze River blocking effect on variations of the Poyang Lake level and floods at annual to decadal scales. The river’s blocking effect diminishes when the lake level is high from receiving large amount of basin discharge albeit a few exceptions to this relationship occurred when river flow also was elevated from receiving large rainfall discharges in upstream areas. Mei et al. (2016a, b) reported that the average contributions of precipitation variation, human activities in the Poyang Lake catchment and TGD regulation to the Poyang Lake recession can be quantified as 39.1, 4.6 and 56.3 %, respectively.

The extend of TGD impacts on the water resources in the Poyang Lake is different for different seasons or drought and flood years as water table and streamflow of the Poyang Lake and the TGD’s impacts on the lower Yangtze streamflow varies greatly during different periods (Gao et al. 2013; Zhang et al. 2012; Li et al. 2016a, Yao et al. 2016; Mei et al. 2016b). The TGD together with the droughts in the Poyang Lake River basin was believed to cause the water level decline in the Poyang Lake in the drier seasons (Lai et al. 2014; Zhang et al. 2014b). Compared to climate variability impacts on the Lake catchment, modifications to Yangtze River flows from the TGD have had a much greater impact on the seasonal dryness (September–October) of the Lake (Zhang et al. 2014b). Guo et al. (2012) found that the Poyang Lake’ seasonal variation follows the TGD’s seasonal impounding and releasing of water. However, the TGD’s seasonal impounding and releasing of water weaken the river forcing on the lake, allowing more lake flow to the river from July to March. Particularly, the low flow of the mid-lower Yangtze River after the operation of the TGD has affected the Poyang Lake greatly (Min and La 2012). The TGD will increase flood risk during the early summer monsoon, in contrast to the original justifications for building the dam due to complex river–lake–groundwater interactions (Nakayama and Shankman 2013).

Although many studies about the impacts of climate change and/or human activities on the water resources of Poyang Lake have been conducted, the knowledge of the impacts of TGD on the water balance of Poyang Lake is limited, which is of great scientific significance in understanding the causes of current shortage of water resource in the Poyang Lake (Guo et al. 2012; Zhang et al. 2014b, 2015). Changes in the Yangtze River discharge caused by the TGD have further altered the interrelationship between the river and Poyang Lake, disturbing the lake basin hydrological processes and water resources. Therefore, to quantify change in river–lake water exchange and its influence on the Yangtze River discharge and the Poyang Lake inflow/outflow is important for estimation of impacts of coupling effects of TGD and droughts on the water balance of the lake. The scientific questions to be investigated in this study include: (1) has the regularity of water balance in the Poyang Lake changed before and after the operation of TGD? (2) Does the TGD and climate change affect the water balance in the Poyang Lake? This study is of importance in further understanding the changes in hydrological processes of the Poyang Lake. This paper will analyze and simulate the change of water quantity using a water balance model for the Poyang Lake, and try to reveal the impacts of climate change and human activities on the lake water balance. In this study, we will analyze the changes of water balance items and their relationship to the operation of TGD based on long-term hydrological and meteorological datasets across the Poyang Lake basin.

## Data and methodology

### Study area and data

**Fig. 1:**
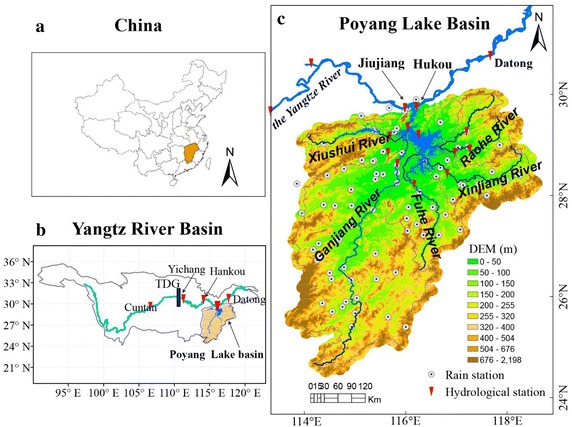
Location of the Poyang Lake river basin and concerned hydro-meteorological stations. **a** Location of China; **b** location of the Yangtze River basin; **c** location of the Poyang Lake River basin

Poyang Lake, China’s largest freshwater lake, is located on the southern bank of the lower Yangtze reach. Poyang Lake is an overflow lake with the characteristic of taking in and sending out water in light of seasonal variations. The water balance at the Poyang Lake is mainly dominated by five main tributary rivers: Ganjiang River, Fuhe River, Xinjiang River, Raohe River and Xiushui River, and several smaller rivers (as shown in Fig. 1). The basin area of the five rivers is 162,200 km^2^, occupying 9 % of Yangtze River basin. In addition, the inflow from the Yangtze River to the Poyang Lake plays an important supplementary role in maintaining the water resources stability of Poyang Lake, especially when the Poyang Lake basin is under a drought situation (Hu et al. 2007). Thus, inflow of the Poyang Lake includes two parts: the inflow from five sub-basins and from the Yangtze River. Streamflow from the Hukou station is regarded as the outflow of Poyang Lake. When the highest lake level at the Hukou hydrological station reaches 22.59 m, the corresponding lake area is approximately 4500 km^2^ with the lake volume of 34 billion m^3^. As the lake level at the Hukou station reaches the lowest of 5.90 m, its corresponding lake area and lake volume are 146 km^2^ and 450 million m^3^, 1/32 and 1/76 of the largest area and volume, respectively (Zhang et al. 2015).

Sixteen daily streamflow and water level stations and 83 daily precipitation stations from 1957 to 2009 were used in this study (Fig. 1; Table 1). Among them, the Hushan station located in Raohe River basin has a 2 years gap at the end of the 1970s which was filled in by Xin’anjiang hydrological model (Zhang et al. 2015). Missing precipitation data has been computed by the back propagation (BP) neural network model. Groundwater data, agriculture water use, industry water use et al. were from the Jiangxi province hydrological yearbook. The scale of DEM is 1:10,000 which is provided by the Changjiang Water Resources Commission of the Ministry of Water Resources and the Landsat is downloaded from: http://glovis.usgs.gov/. The water level from the lake is also taken from the landsat TM satellite images and the survey of lake volume was made by Jiangxi Provincial Water Resources Department.

**Table 1:** Control hydrological stations at the Poyang Lake basins

Tributaries	Station	Area (10^4^ km^2^)	Annual mean streamflow (m^3^/s)
Xiushui R.	Qiujin	9914	265
	Wangjiabu	3548	107
Ganjiang R.	Waizhou	80,948	1986
Fuhe R.	Lijiadu	15,811	379
Xinjiang R	Meigang	15,535	543
Raohe R.	Dufengkeng	5013	147
	Hushan	6374	220
Lake Region	Hukou	162,200	4593

### Methodology

#### Lake water balance equation

To examine the relative magnitude and timing of each of the flows entering and leaving the lake, a water balance model for the lake system was used (Dessie et al. 2015). The equation of water balance of a lake in general form is given by (Unit: mm):

1 $$\frac{\Delta S}{\Delta t} = P_{lake} - E_{lake} + Q_{gauged} + Q_{ungauged} - Q_{out} + \varepsilon$$

where $\frac{\Delta S}{\Delta t}$ denotes change in storage over time, *P*_lake_ is lake areal rainfall, *E*_lake_ is the lake evaporation, *Q*_gauged_ is gauged river inflow and back flow from Yangtze River, Q_ungauged_ is ungauged river inflow, *Q*_out_ is outflow from the Poyang Lake to the Yangtze River, and *ε* represents the uncertainties in the water balance arising from errors in the data and other terms, such as net ground water flux or minor abstractions, which usually cannot be accounted for directly.

#### Calculating lake water balance anomalies

Precipitation plays a major role for water input of the Poyang Lake both directly via the lake surface and indirectly via the water inputs by five main tributary rivers. Too much or too little precipitation can cause significant damage to life and property through floods and droughts (Zhao et al. 2010). In this paper, 68 rain gauge stations are used to calculate the tributaries precipitation and another 15 rain gauge stations are applied to estimate the lake precipitation.

Evaporation is an essential part in the water cycle and it is hard to measure the rate of evaporation from a lake (Zhang et al. 2009). In this paper, the lake evaporation will be estimated by pan evaporation data (E_pan_), and then multiplied by a factor *K*_p_, and *K*_p_ is determined as the rate of reference evaporation (*ET*_ref_) to pan evaporation (*E*_pan_).

The sum of the five tributaries in the Poyang Lake River basin was chosen as the inflow to the Poyang Lake and the Hukou station at the junction of the Poyang Lake and Yangtze River was selected to analyze water exchange between the Poyang Lake and the Yangtze River.

The daily or monthly anomalies of lake water balance were calculated as below:

2 $$S_{2} - \, S_{1} = \Delta S_{2} - \Delta S_{1}$$

where ΔS denotes daily or monthly water balance, S_2_ − S_1_ is the water balance anomalies between adjacent 2 days or months. ΔS_2_ − ΔS_1_ is the difference of lake water balance between the adjacent 2 days or months.

## Results and discussion

### The characteristics of water balance for the Poyang Lake

The water balance items during the past few decades of Poyang Lake have been analyzed. For example, the annual mean precipitation is 1645.6 mm during 1957–2009 in the Poyang Lake River basin with a descending trend from south to north, while the annual mean precipitation for Poyang Lake is 1598.3 mm. The Poyang Lake is a river-communicating lake. The water almost flows from the lake to the Yangtze and sometimes backward from the Yangtze to the Lake. The annual mean lake outflow is 1458.4 × 10^8^ m^3^ which is smaller than that of lake inflow (1543.4 × 10^8^ m^3^). Meanwhile, more droughts and/or floods might occur in this area because the seasonal precipitation varies greatly (Wang et al. 2013b).

**Fig. 2:**
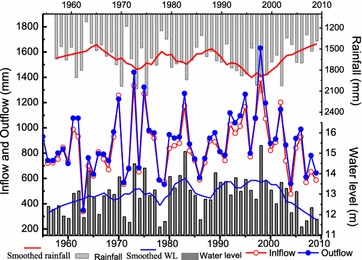
The variability of annual mean streamflow, water level and precipitation in the Poyang Lake basin (in this figure, the same unit (mm/a) was used for both precipitation and streamflow, streamflow comes from five tributary rivers, precipitation is the basin mean)

**Fig. 3:**
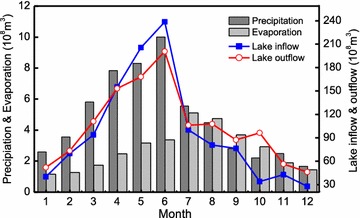
The monthly mean precipitation and evaporation in the region of Poyang Lake and inflow and outflow of Poyang Lake

The long-term tendencies of the annual and seasonal variations of precipitation, inflow, outflow, and water level in the Poyang Lake basin are shown in Fig. 2. It can be seen that annual mean lake inflow corresponds with the annual mean precipitation of the Poyang Lake River basin. The monthly mean precipitation increases from January to June and then decreases from June to December in the Poyang Lake River basin, and a similar pattern is found for the monthly lake inflow and outflow (Fig. 3). Results also indicate that precipitation mainly concentrated in rainy season (AMJ) in the whole basin. The pattern of monthly mean evaporation is similar to that of precipitation except the monthly maximum evaporation appearing in July. From this figure, we find that monthly mean lake inflow is larger than outflow in May and June while lake inflow is smaller than outflow in other months, especially in autumn.

**Fig. 4:**
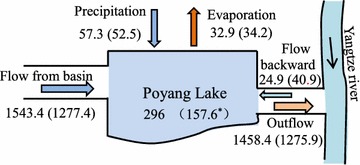
Comparision of water balance items for the Poyang Lake before and after the operation of TGD (*Asterisk* Simulated lake volumn)

**Fig. 5:**
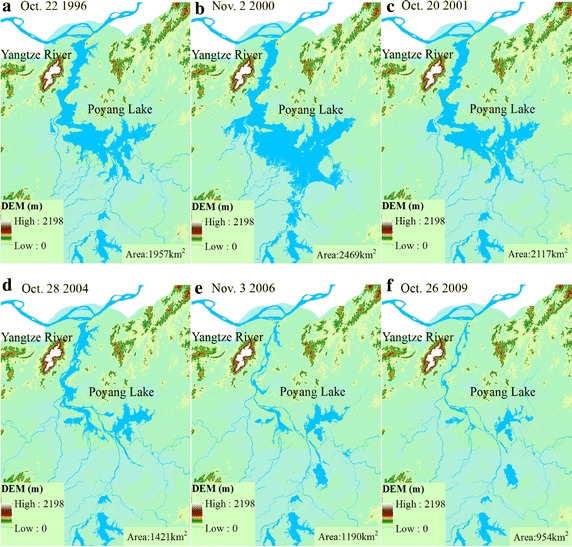
Changes of lake area for the Poyang Lake before and after the operation of TGD extracted from landsat TM images. **a** October 1996; **b** November 2000; **c** October 2001; **d** October 2004; **e** November 2006; **f** October 2009

The water balance of Poyang Lake is mainly dominated by horizontal (lake inflow, outflow) and vertical (lake precipitation, evaporation) balance components (Fig. 4). Generally, positive annual water balance anomalies appeared in the 1970s while negative anomalies occurred in the 2000s. Similar results can also be found in the changes of lake areas. From the changes of lake areas in late October and early November in the Poyang Lake extracted from landsat TM images (Fig. 5), we can find that the lake areas in 2004, 2006, and 2009 are small like a river and the lake areas are only 1421, 1190, and 954 km^2^, respectively, which are far below the average lake area (2181 km^2^) in the year of 1996, 2000, and 2001.

Water inputs from the five sub-basins are particularly important during the major rainy seasons when heavy rainfall produces large surface flows from the sub-basins to the lake (Shankman et al. 2006), which has been changed significantly due to changes in hydro-climatic variables and human activities during the past decades in the Poyang Lake catchment (Xu et al. 2014; Ye et al. 2013; Zhang et al. 2014b, Li et al. 2016c; Awan et al. 2016; Singh et al. 2016). Guo et al. (2008) indicated that the climate effect is dominant in annual streamflow, while land-cover change may have a moderate impact on annual streamflow for the Poyang Lake basin. However, human activities such as construction of reservoirs and irrigation works might increase water consumption (Ye et al. 2009). Wang et al. (2013a) suggested that human activities were the main driving factors for the decline in annual runoff in Luanhe, Chaohe and Zhanghe River catchment, which account for over 50 % of runoff reduction. From Table 2, we can find that water consumption rate varies in the range of 10.02–22.02 % of the total amount of water in the main basin area of Jiangxi province, among which agricultural water consumption is the largest water use in Jiangxi Province and annual total amount water consumption rate varies greatly at the range of 10.02–20.02 %.

**Table 2:** Water consumption of Jiangxi Province in recent years (the data comes from Jiangxi Province Hydrological Yearbook)

Years	Total amount of water (10^8^ m^3^)	Water consumption (10^8^ m^3^)				Water consumption rate (%)
		Agriculture	Industry	Domestic	Others	
1999	1866.00	144.09	45.01	19.21	8.77	11.63
2000	1454.00	141.99	47.50	17.35	10.80	14.97
2001	1523.00	140.21	42.45	18.04	10.23	13.85
2002	1983.26	123.51	46.35	18.94	9.95	10.02
2003	1362.68	93.90	46.75	17.87	13.98	12.66
2004	1034.63	120.74	52.16	18.53	12.09	19.67
2005	1510.1	126.54	51.21	17.76	12.54	13.78
2006	1629.97	128.51	50.57	17.84	8.75	12.62
2007	1112.96	146.82	58.60	20.03	9.42	21.10
2008	1356.16	144.71	59.92	20.52	9.05	17.27
2009	1166.91	168.67	53.18	22.76	12.34	22.02
Pre-TGD	1637.79	128.74	45.61	18.28	10.75	12.42
Post-TGD	1301.79	139.33	54.27	19.57	10.70	17.20

### The impacts of TGD on the water balance of Poyang Lake

Many researchers have reported that the outflow of Poyang Lake is influenced by the water level difference between the Poyang Lake and the main Yangtze River (Guo et al. 2012; Hu et al. 2007; Zhang et al. 2011, 2014b). The fact is that the water surface areas of the Poyang Lake in October before the operation of the TGD are greater than that of the post-TGD period (Fig. 5). Min and La (2012) also thought that the Poyang Lake has been affected greatly by the low flow of the mid-lower Yangtze River after the operation of the TGD. Gao et al. (2013) reported that the river discharge at the Datong gauge during the TGD impoundment period decreased by 18–40 % after the reservoir started full-capacity operations in 2008.

Comparing water balance items between the period of pre-TGD and post-TGD, we find that lake precipitation and lake inflow from basin decrease significantly during the post-TGD periods, 8.3 and 20.5 % of decrease, respectively, compared with those during the pre-TGD period. Meanwhile the evaporation and the backward flow from the Yangtze to the Poyang Lake increase of 4.0 and 64.3 %, respectively (Fig. 4). As a result, the volume of Poyang Lake has dropped significantly. It is noted that the lake outflow has reduced 12.5 % during the two periods of the post-TGD period and the pre-TGD period although the lake precipitation and lake inflow has decreased significantly. This means more water might pour into the Yangtze River from the Poyang Lake when the lake water inputs reduced after the operation of TGD. In other words, negative lake water balance anomalies appear during this period.

**Fig. 6:**
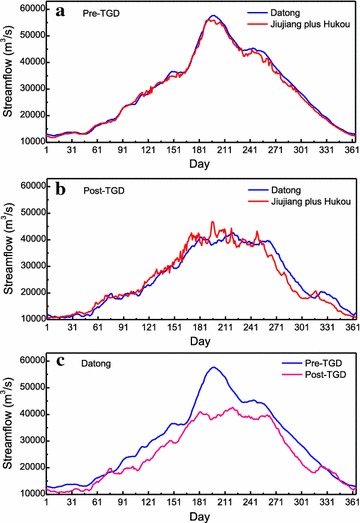
The daily mean streamflow comparison between observed Datong and reconstructed Datong (Jiujiang plus Hukou) before and after the operation of TGD (**a** pre-TGD period, 1988–2003; **b** post-TGD, 2004–2009; **c** the streamflow at Datong before and after TGD)

**Fig. 7:**
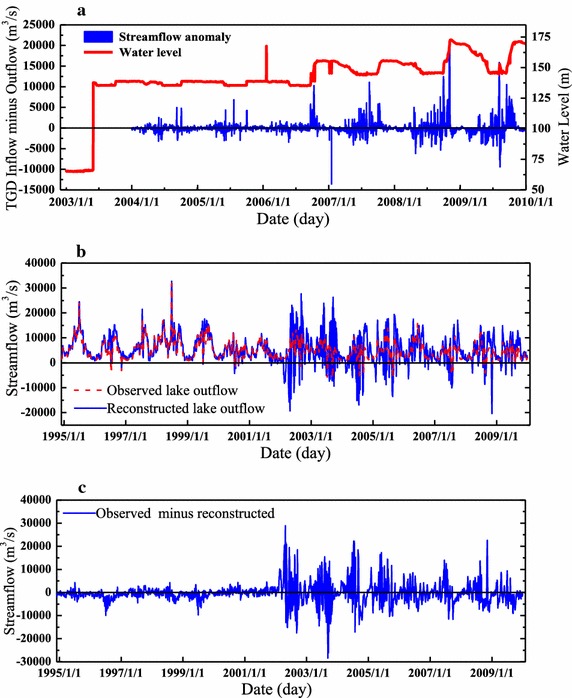
The changes of streamflow before and after the operation of TGD in the Yangtze River (**a** inflow and outflow of TGD and corresponding water level; **b** comparison between the observed Poyang Lake outflow (streamflow of Hukou station) and reconstructed lake outflow (streamflow of Datong minus Jiujiang); **c** observed minus reconstructed lake outflow)

Jiujiang station is very close to Hukou station (32 km upstream), and there is almost no large river flows between Jiujiang and Datong except the Poyang Lake River basin. Zhang et al.(2015) found that there is a good relationship between the water level at the Hukou station and the streamflow at the Datong station during the past several decades. From Fig. 6, we find that the sum of streamflow of Hukou and Jiujiang is almost equal to the streamflow of Datong station before the TGD operation, however, the relationship between them has been changed after the TGD operation. Therefore, the streamflow of Datong minus Jiujiang (reconstructed Poyang lake outflow) is calculated to evaluate the impacts of the main Yangtze River on the Poyang Lake. It can be seen that the reconstructed lake outflow agrees well with the observed lake outflow before the operation of TGD (Fig. 7). However, the reconstructed lake outflow varies more greatly than that of observed lake outflow after the operation of TGD, which indicates that the TGD has made great impacts on the water balance in the Poyang Lake (Fig. 7).

### Coupling effects of TGD and droughts on the water balance of Poyang Lake

**Fig. 8:**
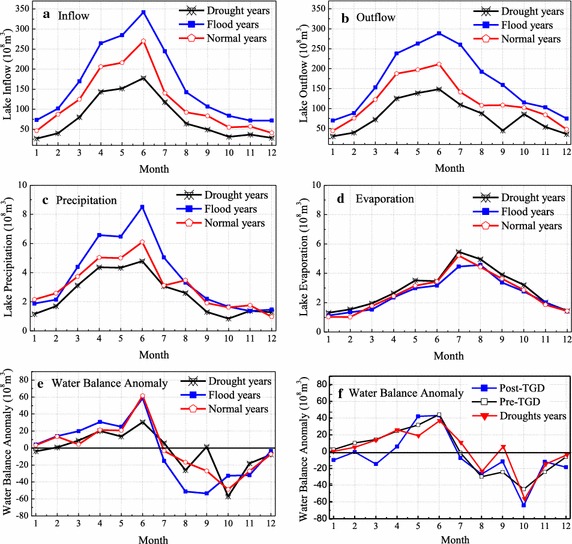
The monthly mean water balance items and water balance anomaly for the normal, drought, flood, and TGD induced years in the Poyang Lake. **a** Lake inflow for drouhgt, flood and normal years; **b** lake outflow for drought, flood and normal years; **c** lake precipitaion for drought, flood and normal years; **d** lake evaporation for drought, flood and normal years; **e** water balance anomaly for drought, flood and normal years; **f** water balance anomaly for post-TGD, pre-TGD and drought years

The monthly mean water balance items for the normal, drought, flood, and post-TGD years in the Poyang Lake has been analyzed to reveal the coupling effects of TGD and droughts on the water balance in the Poyang Lake (Fig. 8). In this paper, the period of 2003–2009 is defined as the post-TGD years when the streamflow influenced by the TGD operation. According to the changes of annual mean streamflow of five tributaries in the Poyang Lake, the years of 1963, 1965, 1968, 1971, 1974, 1978, 1979, 1986, 2004, 2007, 2008 and 2009 are selected as the drought years, while 1970, 1973, 1975, 1983, 1992, 1994, 1995, 1997, 1998, 1999, 2002 are chosen to be the flood years, and the rest of the years are considered as normal years.

We find that the water balance items and water balance anomaly increases from January to June, and then decreases from June to December for the flood, drought and normal years. The variation of water balance anomalies for the drought years is generally similar to that of the post-TGD years. However, the water balance anomalies for the post-TGD years are higher than that of drought years in November, May and June. Also, the water balance anomalies for the post-TGD years are lower than that of drought years in March, April, September and October. It is clear that the water balance anomalies for the normal years are higher than those of drought and post-TGD years in January, February, June, and November while the anomalies are lower than those of the drought and post-TGD years in September. Thus, it can be inferred that the operation of TGD has significantly altered the water balance of Poyang Lake with the TGD releasing and impounding water in the period of April–May and September–October, respectively. Similar results can be found in recent publications, such as Nakayama and Shankman (2013) who analyzed the impacts of TGD on the floods in the Poyang Lake region and found that TGD increases flood risk during the early summer monsoon months against the original justifications for building the dam, relating to complex river–lake–groundwater interactions. Zhang et al. (2014b) demonstrated that the TGD has had a much greater impact on the seasonal (September–October) dryness of the Lake. Zhang et al. (2015) indicated that the combined effect of both the TGD operation and droughts might be the major cause of water scarcity in the Poyang Lake.

Table 2 shows the water consumption in Jiangxi Province before and after the operation of TGD, where we find that total amount of water decreases significantly after the TGD operation while water consumption increases, especially agriculture and industry water consumption. The water consumption rate increases from 12.42 % during the pre-TGD period to 17.02 % for the post-TGD period. From Fig. 6c, the daily mean streamflow at Datong dropped greatly after the operation of TGD, which indicated the impacts of TGD as only one factor. Thus, it can be inferred that the relationship between TGD and lake water balance becomes more complicated during a drought in the Yangtze River basin.

## Conclusions

In this study, the long-term variations of water balance in the Poyang Lake are analyzed with the aim of exploring the possible impacts of TGD on the lake water balance. Some interesting conclusions are obtained as follows:

1. The water balance items have changed greatly after the operation of TGD. The annual mean precipitation has reduced about 4.8 × 10^8^ m^3^, the evaporation has increased 1.3 × 10^8^ m^3^ in the Poyang Lake, while the annual mean streamflow from five tributaries to the Poyang Lake has decreased 266 × 10^8^ m^3^ during the post-TGD period compared to that of pre-TGD period. Although the total amount of water has decreased significantly mainly caused by the droughts in the Poyang Lake River basin in recent years, the water consumption has increased significantly, especially agriculture and industry water consumption.
2. The operation of TGD might have made great impacts on the water balance in the Poyang Lake. The outflow of Poyang Lake might be influenced by the water level difference between the Poyang Lake and the main Yangtze River, therefore, the operation of TGD will increase the river–lake water level gradient which might alter the lake balance by inducing greater discharge into the Yangtze River when the TGD impounding water, especially in October, and negative lake water balance anomalies appear during this period. The water consumption rate in the Poyang Lake river basin increases from 12.42 % during the pre-TGD period to 17.02 % for the post-TGD period.
3. Composite analysis has been used to reveal the coupling effects of drought and TGD on the water balance in the Poyang Lake. The operation of TGD has significantly altered the water balance of Poyang Lake with the TGD releasing and impounding water in the period of April–May and September–October, respectively. The variation of water balance anomalies for the drought years is generally similar to that of the post-TGD years. However, the water balance anomalies for the post-TGD years are higher than that of drought years in November, May and June. Therefore, the droughts and the operation of TGD might cause the river–lake interaction to be more complicated.
